# Novel paradigm for immunotherapy of breast cancer by engaging prophylactic immunity against hepatitis B

**DOI:** 10.1186/s40169-016-0111-8

**Published:** 2016-08-18

**Authors:** Marek Malecki, Chelsea Sabo, Afsoon Foorohar, Xenia Tombokan

**Affiliations:** 1 Phoenix Biomolecular Engineering Foundation, San Francisco, CA USA; 2 University of Wisconsin, Madison, WI USA; 3 National Magnetic Resonance Facility, National Institutes of Health, Madison, WI USA; 4 Sutter Health Center, San Francisco, CA USA; 5 University of Sheffield, Sheffield, UK; 6 Bruker Corporation, Woodlands, TX USA

**Keywords:** Breast cancer, Immunotherapy, Trastuzumab, Cancer, Vaccine, HER-2, *Erb*-*B2*, Mimotope, Hepatitis B

## Abstract

**Background:**

Immunotherapy of patients suffering from the human epidermal growth factor receptor 2 overexpressing (HER-2^+^) breast cancers with the anti-HER-2 antibodies results in increase of the patients’ overall survival. However, no prophylactic vaccine is available against HER-2^+^ breast cancers. Although, prophylactic vaccine for human hepatitis B virus (HBV) is very effective.

**Specific aim:**

The specific aim of this work was to design, synthesize, and test bio-molecules which would engage prophylactic immunity against hepatitis B virus towards killing breast cancers cells.

**Methods and Results:**

By biomolecular engineering, we have created a novel family of biomolecules: antibody (anti-HER-2) × vaccine (HBsAg) engineered constructs (AVEC: anti-HER-2 × HBsAg). These biomolecules were utilized for redirecting, accelerating, and amplifying of the vaccination-induced, prophylactic immunity originally targeted against HBV as therapeutic immunity, newly targeted against HER-2^+^ breast cancers. Treatment of the HER-2^+^ breast cancer cells with AVEC: anti-HER-2 × HBsAg in blood of the patients, vaccinated with HBsAg, rapidly increased efficacy of killing of HER-2^+^ breast cancer cells over that attained with the naked anti-HER-2 antibodies.

**Conclusion:**

Novel antibody-vaccine engineered constructs (AVEC) facilitate redirecting, accelerating, and amplifying of prophylactic, HBV vaccination-induced immunity as immunotherapy (RAAVIIT) of HER-2^+^ breast cancer. We currently streamline this novel therapeutic paradigm into clinical trials of breast and other cancers.

## Background

According to the Centers for Disease Control and Prevention, 220,097 women were diagnosed with the breast cancer—the highest incidence of all female cancers—in the USA in 2011 [1]. Almost 30 % of those cancers overexpressed genes *Erbb*-*B2*; thus diagnosed as HER-2^+^ breast cancers. These cancers are associated with shorter times to relapses, as well as, shorter overall survivals, than those that did not overexpress *Erb*-*B2* (HER2-). These data strongly support administering immunotherapy with antibodies against HER-2 [2–5]. Two domains of the HER-2 receptors are targeted by antibodies currently approved by the FDA: trastuzumab (Herceptin) and pertuzumab (Perjeta) [6, 7]. In combination with the M-phase specific systemic therapeutic-docetaxel, they result in a total survival of more than 4.5 years, compared with 1.5 years achieved 14 years ago [8]. Mechanisms of action include: (I) inhibition of growth by steric inhibition of receptors’ dimerization; (II) antibody-dependent cell-mediated cytotoxicity (ADCC); (III) complement dependent cytotoxicity (CDC) [8–11]. Therefore, efficacy of this immuno-therapy relies heavily upon engaging the patients’ own immune system, as well as repressing resistance [12].

Ideally, the most effective way to reduce such a high incidence of breast cancers would be vaccination. Unfortunately, there are no breast cancer vaccines that are approved by the FDA. The clinical trials with various anti-cancer vaccines resulted in the overall efficacy in the range of 2.6 % so far [13, 14]. This is nowhere near the great efficacy of anti-viral and anti-bacterial vaccines, which are approved by the FDA and recommended by the CDC [15].

Vaccination against various microbials is the greatest achievement of the modern medicine. In particular, the vaccines against hepatitis B virus (HBV) are approved by the FDA and recommended by the CDC: Engerix B and Recombivax [16–18]. Measure of the immune system readiness is production of antibodies by immune cells at the titers above 10.0 mIU/ml. If the antibody titer falls below that aforementioned value, the booster dose quickly reinvigorates the effective immunity. Thanks to this program in the USA, incidence of Hepatitis B declined 82 % over 17 years, i.e., from 8.5 cases per 100,000 population in 1990 to 1.5 cases per 100,000 population in 2007.

In clinical practice, we realized presence of a strange paradox. On one hand we have populations of patients, who are having their entire active immunity system, enhanced due to the FDA approved HBV vaccine, remaining on stand-by. On the other hand, we have populations of patients, who were diagnosed with breast cancers carrying specific molecules HER-2—a potential vaccination target and who would greatly benefit from therapeutic vaccines, but no vaccines are available. This realization prompted our work.

## Specific aim

The specific aim of this project was biomolecular engineering of molecules capable of redirecting, accelerating, and amplifying immunity from the preventive immunity, attained due to HBsAg vaccination against hepatitis B viruses, towards the therapeutic immunity against HER-2^+^ breast cancers.

### Patients

Blood and cancer biopsies were acquired from the ten patients suffering from the advanced breast cancers, from the Acute and Chronic Infection with Hepatitis B virus, and from the healthy volunteers having high titers of antibodies induced by standard HBsAg vaccination. All biopsies were acquired in accordance with the Declaration of Helsinki, Institutional Review Board approval, and with Patients’ Informed Consent (PIC).

## Experimental design

**Fig. 1:**
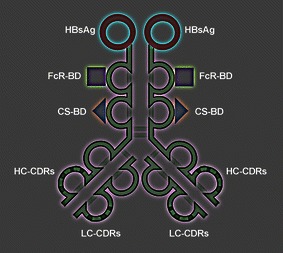
**Functional organization of AVEC: anti-HER-2 × HBsAg**. Antibody x vaccine engineered construct (AVEC): anti-HER-2 x HBsAg consists of the targeting and effector domains. The targeting domains constitute the complementarity determining regions (CDRs) for human epidermal growth factor receptor 2 (HER-2). The effector domains constitute: hepatitis B surface antigen (HBsAg); constant fragment receptor binding domain (FcR-BD); complement system binding domain (CS-BD)

The experimental design is illustrated (Fig. 1). The novel molecule antibody × vaccine engineered construct (AVEC): anti-HER-2 × HBsAg contains the three main effector domains: (1) constant fragment receptor (FcR) binding domain; (2) C1q complement docking domains; (3) hepatitis B surface antigen (HBsAg), which is the HBV VLP. All are guided by the complementarity determining regions (CDRs) within variable fragment domains targeting human epidermal growth factor receptor 2 (HER-2) Fig. 1.

## Methods

### Breast cancer cells

SK-BR-3—human breast cancer cell line was from the American Type Culture Collection (ATCC, Rockville, MD, USA) [19]. It was derived from the pleural effusion in tractu of the advanced, metastatic breast adenocarcinoma. It was overexpressing *Erb-B2* gene to display HER-2 receptors. It was cultured in the base medium: McCoy’s 5a medium (ATCC) (catalog No. 30-2007), which was supplemented with 10 % human serum, 100 units/ml penicillin, 200 mg/ml streptomycin, in the cell culture 75 cm^2^ flask (Corning) (catalog #430641) in the CO_2_ incubators at 37 °C. The medium renewal was performed two times per week. To split, the cultures were briefly rinsed with 0.25 % (w/v) trypsin, 0.53 mM EDTA solution to remove all traces of serum which contains trypsin inhibitor and thereafter treated with that solution. After dispensing into new flasks, they were grown in the same conditions.

MCF-7—human breast cancer cell line was from the ATCC. It was derived from the pleural effusion in tractu of the advanced, metastatic mammary gland epithelial carcinoma. It was not overexpressing the *Erb*-*B2* gene, but was overexpressing estrogen and progesterone receptors [20]. It was used as a negative control foe HER-2 expressing cells. It was cultured in the base medium: Eagle’s minimum essential medium (ATCC) (catalog No. 30-2003), which was supplemented with 10 % human serum, 0.01 mg/ml human recombinant insulin (hrI), 100 units/ml penicillin, 200 mg/ml streptomycin, in the cell culture 75 cm^2^ flask (Corning) (catalog #430641) in the incubators providing 95 % air and 5 % CO_2_ at 37 °C. The medium renewal was two times per week and cultures divided as outlined above.

### Biotechnology of anti-HER-2 and anti-HBsAg antibodies and biosimilars

Biotechnology of anti-HER-2 antibodies synthesis was pursued by adaptation of that originally described, either as new antibodies or as biosimilars to the FDA approved: trastuzumab and pertuzumab as the positive controls [21, 22]. For verification, the DNA plasmid constructs for the anti-HER-2 antibodies variable fragments were imported from the international ImMunoGeneTics (IMGT, Paris, F, EU) antibody sequences’ bank [23, 24].

Briefly, in the first technology, the B cells were isolated from the blood of patients suffering from the cancers. White blood cells (WBC) were isolated using Ficoll-Hypaque technique. The total mRNA was isolated using Trizol reagent (Molecular Research Center, Inc. Cincinnati, OH). The cDNA was generated using random hexamers (Intergrated DNA Technologies, Coralville, IA) and reverse transcriptase (Promega, Madison, WI) in reactions involving denaturing RNA at 70 °C followed by reverse transcription carried at 42 °C for 15 min. The cDNA quality was tested by the polymerase chain reaction (PCR) of beta actin and GAPDH as reference genes with the commercially available primers (ABI, Foster City, CA). For amplification of coding sequences of the variable fragments, the primers sets were designed using the Kabat database. They were synthesized on 380A DNA Synthesizer (ABI, Foster City, CA). The variable fragments were amplified with polymerase chain reaction using the mix of the generated cDNA, the synthesized primers, dNTPs, and Taq DNA polymerase (Hoffmann–La Roche, Basel, Switzerland) on the Robocycler (Stratagene, San Diego, CA) or Mastercycler (Eppendorf, New York, NY). The blunt ended amplicons were inserted into the plasmid coding for the constant regions of the human antibodies with sequences imported from the Gene Bank. The DNA plasmid construct also contained metal binding domains capable of chelating superparamagnetic and fluorescent metals as detailed [21]. After electroporation of plasmids into fresh B cells or cultured human myelomas, they were propagated and expressed as described [22].

For selection and in vitro evolution, the HER-2 receptors were extracted from the human HER-2^+^ breast cancer cells by immunoprecipitation of rapidly frozen, crushed, thawed, and lyophilized. Alternatively, the mimotopes of HER-2 were manufactured. Both served as the molecular baits and references for validation of antibodies.

Alternatively, biotechnology of trastuzumab and pertuzumab biosimilars was crafted on such a way that the coding sequences for the anti-HER-2 antibodies’ variable fragments were imported from the IMGT. These sequences were synthesized, cloned, expressed, and modified on the same way as the newly developed anti-HER-2 antibodies as described above.

For generating of anti-HBsAg antibodies the B cell were acquired from the patients suffering from the Acute and Chronic Hepatitis B. The protocol was identical to that published (22). Dane particles, isolated from the patients’ blood by PEG gradients precipitation or from liver biopsies by CsCl gradient centrifugations, were rapidly frozen, lyophilized and stored. Alternatively, HBsAg were produced in human hepatoma cells transfected with plasmid DNA. Prior to selection, during in vitro evolution, they were reconstituted with buffer and served as the molecular baits. They also served as the negative controls for anti-HER-2 antibodies.

The metal binding domains of the antibodies were saturated with Gd, Tb, Ru, Ni, Co, or Eu or linked with Au coated Fe_3_O_4_ [Au(Fe_3_O_4_)] nanoparticles. The specificity and sensitivity were determined based upon elemental spectra acquired with EDXS (Noran, Middleton, WI, USA), EELS (Zeiss, Oberkochen, D, EU), or TRXFS (Bruker AXS, Fitchburg, WI, USA). The fluorescent properties were measured with the RF-5301PC spectrofluorometer (Shimadzu, Tokyo, Japan). The magnetic relaxivities were measured on the DMX 400 WB or AVANCE II NMR spectrometers (Bruker Optics, Dallas, TX, USA). Specificity and sensitivity towards specific domains of the receptor were tested by cross-blocking (Table 1).

**Table 1:** Cross-blocking of AVEC: anti-HER-2 × HBsAg

	Trastuzumab	Anti-HER-2_001_	Anti-HER-2_004_	Anti-HER-2_001_ × HBsAg	Anti-HER-2_004_ × HBsAg	Anti-HBsAg
Trastuzumab	+	+	−	+	−	−
Anti-HER-2_001_	+	+	−	+	−	−
Anti-HER-2_004_	−	−	+	−	+	−
Anti-HER-2_001_ × HBsAg	+	+	−	+	−	−
Anti-HER-2_004_ × HBsAg	−	−	+	−	+	−
Anti-HBsAg	−	−	-	−	−	+

### Biotechnlogy of HBsAg

HBsAg was isolated from the patients suffering from Acute and/or Chronic Hepatitis B: either from the blood by PEG fractionations or from the liver biopsies by CsCl gradient centrifugation.

To assure exact immunogenic compatibility with the immunity induced by vaccinations with the FDA approved HBsAg, which were produced in yeast, the HBsAg in this project were also generated in yeast as originally described [25, 26]. Biotechnology of the recombinant HBsAg was pursued based upon the published DNA coding sequence. Hepatitis B virus like particles (VLP) were initially synthesized in yeast—*Saccharomyces cerevisiae* as originally described. In particular, the expression plasmid pHBS-16 included the HBsAg surface antigen (HBsAg) controlled by the yeast alcohol dehydrogenase (*ADHI*) promoter through introduced by EcoRI restriction sites into the DNA construct of the pBR322 plasmid. That followed by yeast replication origin, yeast *trp1* gene. This biotechnology was later modified to be pursued in *Pichia pastoris* [27]. Briefly, yeast cultures of *Pichia pastoris* were grown at 30 °C in rich medium (YPD; 1 % yeast extract, 2 % bactopeptone, 2 % glucose) initially and shifted either to synthetic media (YNM, 0.67 % yeast nitrogen base supplemented with 0.5 % (v/v) methanol) for immunoprecipitation and immunofluorescence experiments, or to mineral media (MMOT, 0.2 % (v/v) oleate and 0.02 % (v/v) Tween-40) for fractionation studies.

All the protocols’ products—HBsAg VLPs were referenced and validated to the FDA approved and the CDC recommended Engerix B and Recombivax and standard clinical diagnostics.

### Biotechnology of fluorescent and superparamagnetic mimotopes

Design of HER-2 cyclic mimotopes was initiated by importing the DNA from the GenBank and in vitro translation into amino acid sequences or direct amino acid sequences from SwissProt into the Peptide 3D or LaserGene software. That followed by determination of surface displayed domains. Further analysis led to selection of the most likely immunogenic domains. The 12–40 amino acids long sequences were selected. The amino acid sequences were exported directly into the program of the peptide synthesizer (ABI, Foster City, CA). The selected sequences were altered by introducing glycine linkers with terminal cysteines at both amino and carboxyl terminus of the peptide designs. The designed peptides were synthesized as linear on the peptide synthesizer. After detachment from the cartridges, the peptides were converted into cyclics by means of the cysteines. The synthetic products—HER-2 mimotopes were selected on the high pressure liquid chromatography columns.

The specificity of the mimotopes was validated by binding to trastuzumab and ant-HER-2 antibodies with the aid of MACS or FACS.

### Biotechnology of anti-HER-2 × HBsAg biomolecular clusters

The synthetic anti-HER-2 antibodies and synthetic HBsAg VLPs were linked with heterospecific, bifunctional linker—sulfo-m-maleimidobenzoyl-*N*-hydroxysuccinimide ester (SMBS) after adapting the protocol [28]. Briefly, the anti-HER-2 antibody was dialyzed against 0.15 M sodium chloride, 0.1 M sodium phosphate, at pH 7.2. Sulfo-MBS stock in DMSO was added to this solution up to the final 2 % w/v concentration to assure at least 80x molar excess. After 1 h at room temperature, the reaction solution was rapidly applied to desalting columns. Performing chromatography with the 0.15 M sodium chloride, 0.1 M sodium phosphate, at pH 7.2 carrier solution was followed by pooling the activated anti-HER-2 antibodies 1 ml fractions. To this solution, the synthetic HBsAg diluted in the same carrier solution was promptly added to assure 1:1 ratio. The reaction continued for 1 h at room temperature. The effective anti-HER-2 × HBsAg clusters were isolated by chromatography.

The specificity of the anti-HER-2 × HBsAg to label HER-2 receptors was validated by FCM on cells and by NMR and XRFS on mimotopes. The specificity of the anti-HER-2 × HBsAg to attract immune response was validated by labeling with anti-HBsAg antibodies rendered fluorescent for FCM or superparamagnetic for NMR [21].

### Immunoblotting and immunoprecipitation

The cells and tissues were either frozen crushed in the rapid controlled rate freezer (NSF grant support to MM).or native disintegrated with ultrasonicator (Branson Ultrasonic, Danbury, CT, USA). After being homogenized within the sample buffer they were either stored in liquid nitrogen or lyophilized. They were electrophoresed in the native buffer (Invitrogen, Carlsbad, CA, USA). They were vacuum- or electro-transferred onto the PVDF membranes (Amersham, Buckinghamshire, UK, EU). The membranes carrying the transferred proteins were first soaked within human serum and thereafter labeled with the bioengineered, biosimilar, and referenced anti-HER-2 antibodies. The anti-HBsAg isotype antibodies served as the controls. The images of the blots were acquired and quantified with Fluoroimager (Molecular Dynamics, Sunnyvale, CA, USA) or Storm 840 (Amersham, Buckinghamshire, UK, EU).

The anti-HER-2 and anti-HBsAg antibodies were rendered magnetic or fluorescent by conjugating Au coated Fe_3_O_4_ nanoparticles or fluorochromes. The sera and liver biopsies’ homogenates were mixed with these superparamagnetic antibodies. The targeted molecules rendered fluorescent were pulled out by the means of 1.5T magnet. The intensity of fluorescence was measured on the spectrofluorometer.

### Fluorescent antibodies. Fluorescent, activated cell sorting. Flow cytometry. Multiphoton fluorescence spectroscopy

 SK-BR-3, MCF-7, and patients’ breast cancer cells were labeled with the fluorescent antibodies. They were sorted on the Calibur, Vantage SE, or Aria (Becton–Dickinson, Franklin Lakes, NJ, USA). The antibodies were dissolved and all washing steps carried in phenol-free, Ca^+^/Mg^+^ free, PIPES buffered saline solution, supplemented with 20 mM glucose, 10 % human serum. Sorting was performed on Aria, Calibur, Vantage SE (Becton–Dickinson, Franklin Lakes, NJ, USA) with the sheath pressure set at 20 lb per square inch pressure and low count rate. The sorted batches were analyzed on Calibur or Aria using FACS Diva software or on the FC500 (Beckman-Coulter, Brea, CA, USA). For the measurement of the fluorescently labeled cells, these settings were tuned at the maximum emission for the Eu chelated antibody at 500 V with references to isotype antibodies and non-labeled cells. This assured the comparisons between populations of cells labeled with multiple antibodies without changing the settings on PMTs.

The fluorescently labeled cells or tissues were imaged with the Axiovert (Zeiss, Oberkochen, D, EU) equipped with the Enterprise argon ion (457, 488, 529 nm lines) and ultraviolet (UV) (364 nm line) lasers; Odyssey XL digital high-sensitivity with instant deconvolution confocal laser scanning imaging system operated up to 240 frames*/*s (Noran, Madison, WI, USA), and the Diaphot (Nikon, Tokyo, Japan) equipped with the Microlase diode-pumped Nd:YLF solid state laser (1048 nm line).

### Superparamagnetic antibodies. Nuclear magnetic resonance spectroscopy. Magnetic activated cell sorting

SK-BR-3, MCF-7, and the patients’ breast cancer cells were labeled with the super paramagnetic anti-HER-2 and anti-phosphatidylserine (anti-PS) antibodies propidium iodide, and bisbenzimide (40). The antibodies were dissolved and all washing steps carried in phenol-free, Ca^+^/Mg^+^ free, PIPES buffered saline solution, supplemented with 20 mM glucose, 10 % human serum. The aliquots were dispensed into the magnetism-free NMR tubes (Shigemi, Tokyo, Japan). The relaxation times T1 were measured in resonance to the applied pulse sequences on the NMR spectrometers: DMX 400 WB or AVANCE II NMR (Bruker, Billerica, MA) or the Signa clinical scanners (GE, Milwaukee, WI, USA).

The superparamagnetic antibodies were also used to isolate the labeled cells from the solution. The cancer cells labeled with the superparamagnetic antibodies were isolated on the magnetic, activated cell sorter operated at 1.5T (NSF grant support to MM).

### Elemental-tags modified antibodies. Energy dispersive X-ray spectroscopy. X-ray reflection fluorescence spectroscopy

The samples, which were cryo-immobilized, presented the life-like antigenicity and supramolecular organization. Elemental analyses were pursued by EDXS and XRFS as described [21]. The field emission, scanning transmission, electron microscope FESTEM HB501 (Vacuum Generators, Kirkland, WA, USA) was equipped with the energy dispersive X-ray spectrometer (EDXS) (Noran, Middleton, WI, USA) and post-column electron energy loss spectrometer (EELS) (Gatan, Pleasanton, CA). The cryo-energy filtering transmission electron microscope 912 Omega was equipped with the in-column, electron energy loss spectrometer (EELS) and the energy dispersive X-ray spectrometer (EDXS) (Zeiss, Oberkochen, D, EU). The cryo-energy filtering transmission electron microscopes 410 and 430 Phillips were equipped with the post-column, electron energy loss spectrometers (EELS) and the energy dispersive X-ray spectrometer (EDXS) (Noran, Middleton, WI, USA). The field emission, scanning electron microscope SEM1530 (Zeiss, Oberkochen, D, EU) was equipped with the energy dispersive X-ray spectrometer (EDXS) (Noran, Middleton, WI, USA). The field emission, scanning electron microscope 3400 was equipped with the energy dispersive X-ray spectrometer (EDXS) (Hitachi, Tokyo, Japan). The S2 Picofox XRFS spectrometer was equipped with a molybdenum (Mo) X-ray target and the Peltier cooled Xflash Silicon Drift Detector (Bruker AXS, Fitchburg, WI, USA). Scan times ranged up to 1000s. The ICP standard of 1000 mg/l of mono-element Gallium or Gadolinium (CPI International, Denver, CO, USA) was added to 500 μl of each sample to the final concentration of 10 mg/l. Instrument control, data collection, and analysis were under the SPECTRA 7 software (Bruker AXS, Fitchburg, WI, USA).

### Antibody dependent redirected-vaccination-induced immunity toxicity (ADRIT)

To study collective killing effects of the anti-HER-2 and anti-HER-2 × HBsAg upon the breast cancer cells, the patients’ cell and serum fraction described below were pooled making erythrocytes-free blood (EFB). Anti-HER-2 and anti-HER-2 × HBsAg were added to the EFB. So were, anti-HBsAg, anti-HPV, anti-HSV, EGFR1, and isotype antibodies as the controls. The incubation with the antibodies continued at the 37 °C incubators. The labeling continued for 1–24 h. It was terminated by washing with the cold buffer.

To quantify the numbers of killed cells by flow cytometry (FCM) and fluorescent activated cell sorting (FACS) the samples were stained with propidium iodide (PI) (Sigma-Aldrich, Milwaukee, WI, USA) used at 50 µg/ml. To determine the numbers of apoptotic cells, they were labeled with anti-phosphatidylserine antibodies.

### Antibody dependent cell cytotoxicity (ADCC)

To study toxicity to the breast cancer cells caused by the patients’ cytotoxic cells—the effectors triggered by the anti-HER-2 antibodies, the peripheral blood mononuclear cells were separated from the blood on Ficoll-Hypaque density gradients. The cells were washed by three cycles of spinning down and suspending in the PBS at pH 7.3. They were rendered fluorescent by adding the stock solution of the DiI membrane dye (Molecular Probes, Inc., Eugene, OR, USA) in DMSO for 10 min at 26 °C. Small aliquots were washed with the buffer and the cells quantified on FCM as the way to determine the effector to target cells’ ratios (ETR). These ratios varied: 10:1, 50:1, and 100:1. Incubations lasted 1–7 h in a 37 °C, 5 % CO_2_ incubator.

The numbers of killed cells were determined due to staining with the PI at 50 µg/ml and of surviving cells from the DiO staining counts and thymidine incorporation.

### Complement dependent cytotoxicity (CDC)

To study toxicity to the breast cancer cells caused by the patients’ complement system—the effector, the serum was separated by gentle centrifugation from the freshly drawn blood. It was supplemented with the anti-HER-2 and anti-HER-2 × HBsAg. Incubations lasted 1–7 h in a 37 °C, 5 % CO_2_ incubator. The numbers of killed cells were determined due to staining with the PI at 50 µg/ml and of surviving cells from the DiO staining counts and thymidine incorporation.

### Statistical analysis

All the measurements were run in triplicates for each sample from six patients. The numbers were analyzed and displayed using GraphPad software (GraphPad Software, Inc, La Jolla, CA). Data were presented as mean of standard error of the mean (SEM). Statistical significance was calculated by *t* test for two groups.

## Results

### Sensitivity and specificity of AVEC: anti-HER-2 × HBsAg in targeting of breast cancer cells and human breast epithelial (HBE) cells

The diagram of the antibody × vaccine engineered construct (AVEC): anti-HER-2 × HBsAg—the novel synthetic bio-molecule is shown (Fig. 1). It consists of the two main sets of domains: targeting: anti-HER-2 antibody and effector: HBsAg—vaccines; FcR-BD–Fc receptor binding domain; and CD-BD—complement system binding domain. Therefore, one of the most essential factors for attaining high efficacy of targeted immunotherapy is its specificity and sensitivity in targeting the HER-2 receptor and HBsAg.

**Fig. 2:**
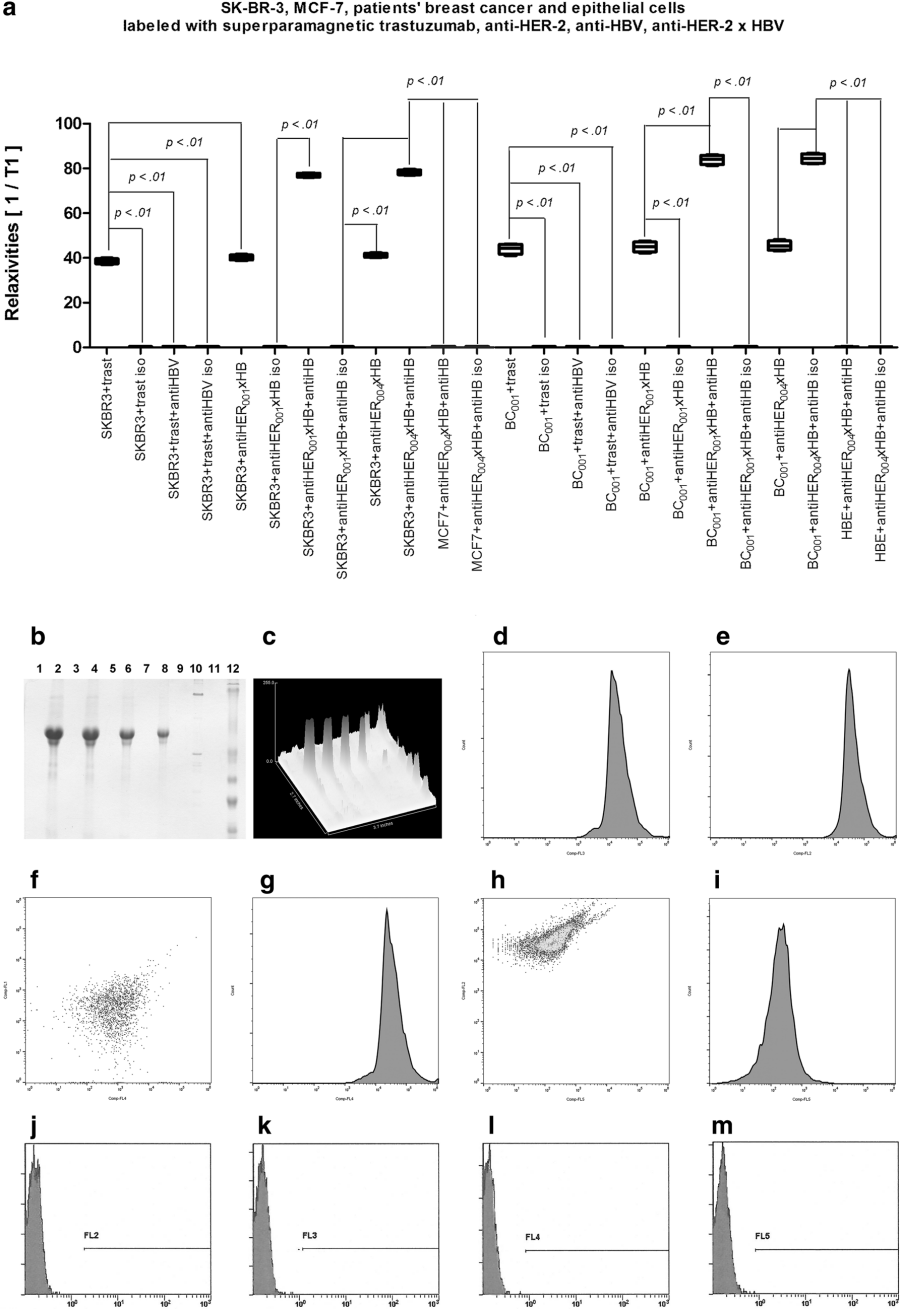
**Sensitivity and specificity of anti-HER-2 × HBsAg**. **a** The breast cancer cells were labeled with trastuzumab, anti-HER-2_001_ × HBsAg, anti-HER-2_004_ × HBsAg, anti-HBsAg (anti-HB) and relevant isotype antibodies (iso) as indicated, followed by secondary superparamagentic antibodies. Measured changes in relaxivities reflect specificity and sensitivity of labeling. All experiments were conducted three times. The data presented are representative for all. The SK-BR-3 and the patients’ breast cancer cells (BC001 - the data for the patient 001 are representative to all 10 patients) cells were heavily labeled with trastuzumab, anti-HER-2 antibodies, and anti-HER-2 × HBsAg constructs. The MCF-7 and the patients’ human breast epithelial cells (HBE) were not labeled at the statistically significant relaxivity change. The isotype antibodies did not label the breast cancer and healthy cells. **b** Sera of patients with high titers of anti-HBsAg antibodies were rapidly cryoimmobilized, crushed, thawed, immunoprecipitated with superparamagnetic molecular baits as indicated, released into electrophoresis, and immunoblotted. Lanes’ of superparamagnetic immunoprecipitation labels. *1*. Immuno-naïve patient, not immunized, not infected, tested with the clinical diagnostics. *2*. Vaccinated patient tested with anti-HER-02_001_ x HBsAg; *3* Vaccinated patient tested with isotype antibody; *4*. Vaccinated patient tested with anti-HER-02_004_ x HBsAg; *5*. Vaccinated patient tested with isotype antibody; *6*. Vaccinated patient tested with Engerix; *7*. Vaccinated patient tested with isotype antibody; *8*. Vaccinated patient tested with Recombivax; *9*. Vaccinated patient tested with isotype antibody; *11*. Vaccinated patient tested with anti-HBsAg isotype antibody *10*, *12* molecular weight standards. Three experiments provided the same data. All molecular baits used in this study were pulling out the same anti-HBsAg molecule as determined by Hepatitis B clinical diagnostic assays. (**c**) The blots from (**b**) were quantified at high sensitivity and revealed only anti-HBsAg antibodies in the patients sera and no other molecules immunoprecipitated and labeled. (**d**–**m**) The breast cancer cells were labeled with trastuzumab (**d**), anti-HER-2_004_ × HBsAg (**e**) mix of both tagged with different flurochromes, (**f**) anti-HER-2_001_ × HBsAg followed by anti-HBsAg fluorescent antibodies (**g**) or after initial labeling with anti-HER-2_001_ × HBV, the cells were labeled with trastuzumab. (**h**, **i**) Isotype antibodies for trastuzumab (**j**) anti-HER-2_001_ (**k**) anti-HER-2_004_ (**l**) anti-HBsAg (**m**) did not reach statistically significant counts of the labelled cells

We measured sensitivity of detection of HER-2 on cancer cells by labeling cells with superparamagnetic antibodies and measuring relaxivities by nuclear magnetic resonance (NMR) (Fig. 2a). For this purpose, the SK-BR-3, MCF-7, patients’ breast cancer cells, and human breast epithelial cells were labeled with trastuzumab, anti-HER-2_001_, anti-HER-2_004_, anti-HER-2_001_ × HBsAg, and anti-HER-2_004_ × HBsAg and anti-HBsAg. As the control, the MCF-7 cells, which do not overexpress HER-2, were labeled with the same antibodies. As the control, these cells were also labeled with the isotype antibodies. The measurements revealed statistically significant differences between the HER-2^+^ SK-BR-3 and patients’ HER-2+ breast cancer cells, which were heavily labeled with therapeutics: trastuzumab, anti-HER-2_001_, anti-HER-2_004_, anti-HER-2_001_ × HBsAg, and anti-HER-2_004_ × HBsAg versus the HER-2^−^ MCF-7 and human breast epithelial cells, which were practically not labeled. These measurements revealed also statistical differences between these therapeutics and the isotypes. Therefore, we validated efficient targeting of the HER-2^+^ SK-BR-3 and the patients' HER-2+ breast cancer cells (the measurements included are representative for all 10 patients’ biopsies studied) breast cancer cells by the anti-HER-2 × HBsAg.

We measured specificity of targeting of the HBsAg by vaccinated and infected patients’ antibodies, while measuring the concentration of this immunogen immuno-precipitated, which was electrophoresed, immunoblotted (Fig. 2b, c). All molecular baits used in this study were pulling out the same 150 kDa molecule, which was recognized by clinical diagnostics for Hepatitis B. By quantitative high gain scanning of the blots, we were able to determine that there were no other molecules pulled out from the sera. The same experiments conducted on the sera of not immunized patients resulted in empty lanes (Fig. 2b, c). Therefore, we concluded that anti-HER-2_001_ × HBsAg and anti-HER-2_004_ × HBsAg were targeted the same molecule anti-HBsAg.

Finally, we determined specificity and sensitivity of the HER-2 domains’ targeting by flow cytometry (Fig. 2). Moreover, we performed tests of cross-blocking (Table 1). While labeling with anti-HER-2_001_ × HBsAg interfered on the statistically significant level with trastuzumab, labeling with anti-HER-2_004_ × HBsAg did not. Therefore, we concluded that these antibodies: trastuzumab and anti-HER-2_001_ × HBsAg target the same, but anti-HER-2_004_ the different domains on the HER-2 receptors.

### Mechanism of action of anti-HER-2 × HBsAg in breast cancer cells’ killing

**Fig. 3:**
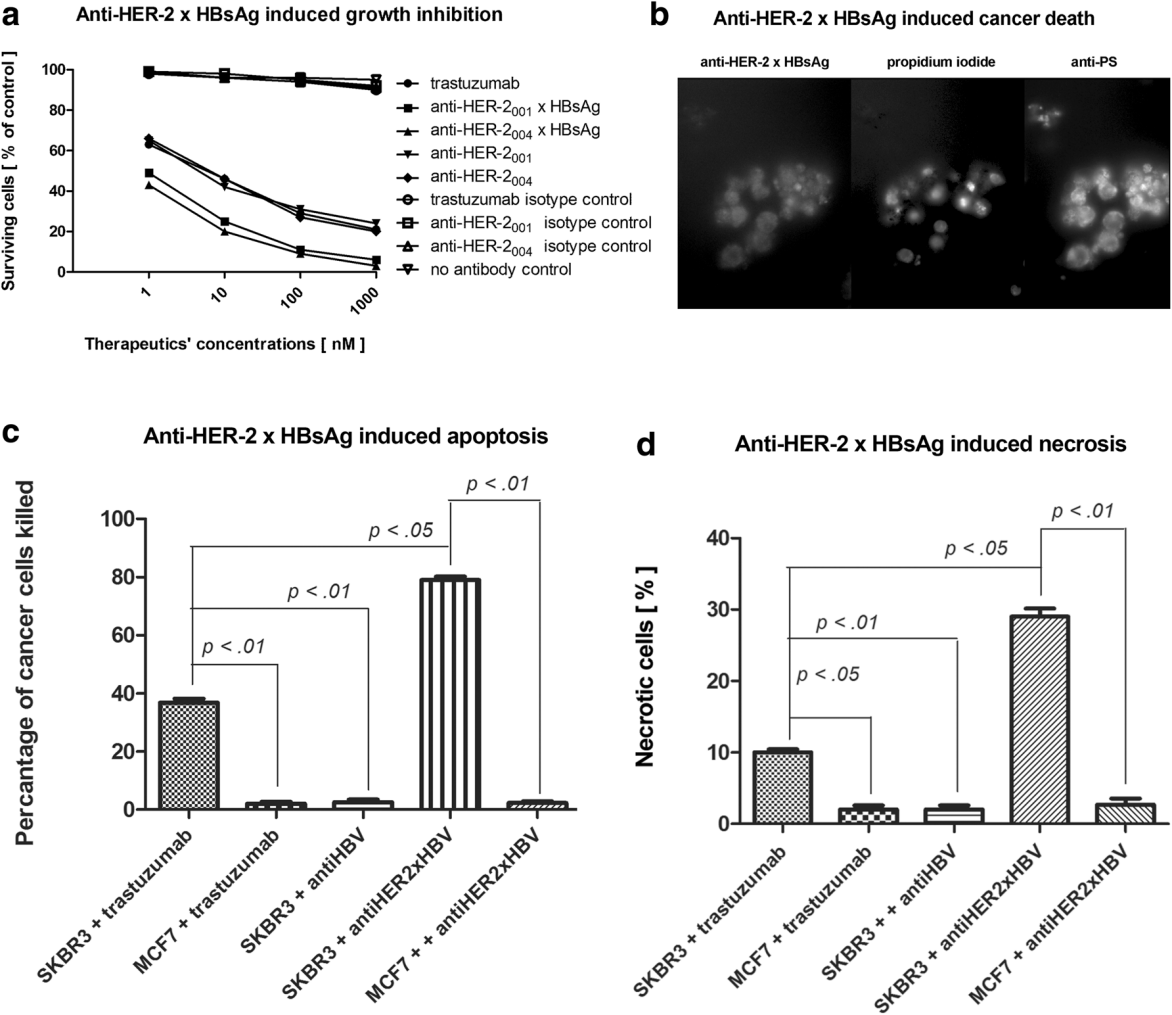
**Mechanism of action of AVEC: anti-HER-2 × HBsAg**. **a** SK-BR-3 breast cancer cells were labeled for 1–7 h at 37 °C with 0.3 mg/ml trastuzumab or anti-HER-2 × HBsAg in full human sera from the HBV—vaccinated patients keeping 10.0 IU/ml anti-HBV. That was followed by studying growth inhibition through thymidine incorporation, **b** The breast cancer cells from the patient with metastatic HER-2^+^ breast cancer were treated as in (**a**) including fluorescent AVEC: anti-HER-2 x HBsAg, labeling with anti-phosphatidylserine (anti-PS) to detect apoptosis or propidium iodide (PI) to detect necrosis. These tests were repeated three times. This route of MOA is representative for all 10 patients’ breast cancer cells. After 1 h only a few cells were showing signs of chromatin collapse. After 6 h most of the cells, which were marked as apoptotic by anti-PS (**c**) were also necrotic. Almost 40 % of the cancer cells were apoptotic due to treatment. That more than doubled due to the treatment with anti-HER-2_001_ × HBsAg or anti-HER-2_004_ × HBsAg. Nearly 10 % were determined necrotic as the result of the treatment with trastuzumab (**d**). The percentage of the necrotic cells due to the treatment with anti-HER-2_001_ × HBsAg or anti-HER-2_004_ × HBsAg more than tripled over that attained with trastuzumab

The three main mechanisms, attributed to the therapeutic efficacy of trastuzumab and pertuzumab, are: growth inhibition, apoptosis, and necrosis. We performed the relevant tests in relation to our AVEC: anti-HER-2 × HBsAg (Fig. 3).

Treatment of the breast cancer cells with increasing concentrations of trastuzumab, anti-HER-2 biosimilars, and the novel anti-HER-2_001_ × HBsAg biomolecules was followed by pulsing with thymidine marked with tritium. Growth inhibition was calculated as percentage of surviving cells compared to non-treated cells as the control (Fig. 3a). Growth inhibition was attained at much lower concentrations, when the cells were treated with anti-HER-2_004_ × HBsAg, over that attained with trastuzumab.

Trastuzumab and both new biomolecular clusters triggered apoptosis (Fig. 3b). The extent of apoptosis progressed rapidly, but the anti-HER-2_001_ × HBsAg and anti-HER-2_004_ × HBsAg induced apoptosis in much greater percentage than trastuzumab (~40 %). In all cases, this percentage was statistically much higher than in the MCF-7—HER-2^−^ cells treated on the identical way (Fig. 3c). It was also statistically significantly higher than SK-BR-3 cells treated with the isotype antibody.

Trastuzumab and both new biomolecular clusters of anti-HER-2 × HBsAg caused necrosis (Fig. 3d). At the initial stages of apoptosis, many cells were only showing outer-membrane display of phosphatidylserine, but were not permeable for PI. At the more advanced stages they were becoming leaky; thus adding to necrotic counts. Both clusters of anti-HER-2 × HBsAg caused massive necrosis of the SK-BR-3 and the patients’ HER-2^+^ breast cancer cells in the percentages, which were statistically significantly much higher over those inflicted by trastuzumab.

### Factors affecting immunotherapeutic efficacy of anti-HER-2 × HBsAg

**Fig. 4:**
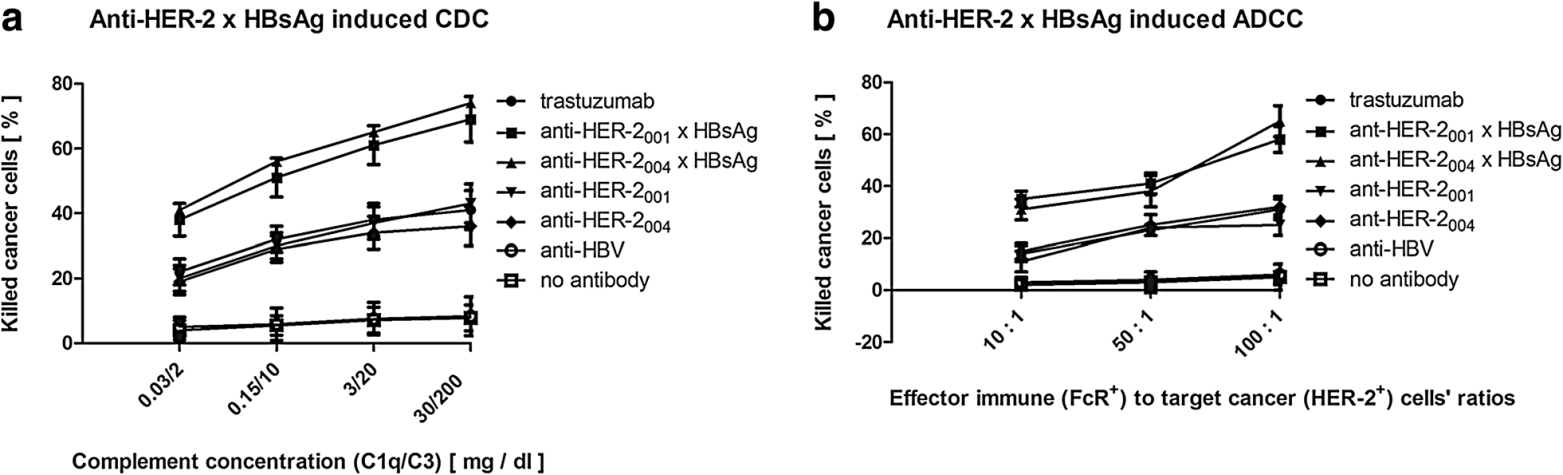
**Factors affecting immunotherapeutic efficacy of anti-HER-2 × HBsAg**. (**a**) SK-BR-3 , MCF-7, and the patients’ HER-2^+^ breast cancer cells were treated with trastuzumab, biosimilar anti-HER-2, anti-HBV, and anti-HER-2 × HBsAg in erythrocyte-free blood, in which concentrations of the complement system were adjusted according to measuring of C1q and C3 at 37 °C. The experiments were concluded by labeling of the cells with propidium iodide. Necrotic cells were counted by flow cytometry. The experiments were repeated three times. Increasing concentrations of complement system components resulted in increased efficacy of the breast cancer cells killing. The novel clusters anti-HER-2_004_ × HBsAg and anti-HER-2_001_ × HBsAg more than doubled the efficacy of the CDC over trastuzumab and anti-HER-2 biosimilars. **b** SK-BR-3, MCF-7, and the patients' breast cancer cells were treated with trastuzumab, anti-HER-2, anti-HBV, and anti-HER-2 × HBsAg is erythrocyte-free blood, in which the number of the immune cells was adjusted in relation to the number of breast cancer cells. Measurements were pursued as in (**a**). Increasing the ratio of the effector immune cells to target cancer cells resulted in proportional increase in efficacy of the breast cancer cells’ killing. The novel clusters anti-HER-2_004_ × HBsAg and anti-HER-2_001_ × HBsAg more than tripled the efficacy of the ADCC over trastuzumab and anti-HER-2 biosimilars

The processes of breast cancer cells’ deaths are triggered by the specific elements of the patients’ immune system: humoral and cellular. We aimed at defining the main factors triggering them. In particular, we were focused on effects of complement concentrations and effector cells to target cells ratios (Fig. 4a, b).

Concentrations of the complement systems’ components (CS) determine the patients’ ability to fight cancer by complement dependent cytotoxicity (CDC). Concentrations of the complement systems’ components in our tests were adjusted within the ranges of healthy adults (Fig. 4a) Our measurements revealed that increasing the concentrations of the C1q and C3 resulted in the statistically significant increase in the efficacy of the HER-2^+^ SK-BR-3 and the patients’ HER-2^+^ breast cancer cells’ killing by trastuzumab and anti-HER-2 antibodies as compared to labeling with the isotype antibodies or labeling of the HER-2^−^ MCF-7 cells. The efficacy was statistically significantly much higher, when the HER-2^+^ SK-BR-3 and the patients' HER-2^+^ breast cancer cells were treated with anti-HER-2_001_ × HBsAg, and anti-HER-2_004_ × HBsAg.

Numbers of natural killer cells and cytotoxic lymphocytes in the patient’s circulation determine this patient’s ability to execute antibody dependent cell cytotoxicity (ADCC). The numbers of the immune cells were adjusted to clinical lab values. Trastuzumab and our anti-HER-2 biosimilar antibodies caused the cancer cells’ deaths through ADCC already at the ratio of 10:1, but with no statistical difference between them (Fig. 4b). Anti-HER-2_001_ × HBsAg, and anti-HER-2_004_ × HBsAg inflicted massive deaths of breast cancer cells, which were at statistically significantly much higher levels, than those inflicted by trastuzumab and anti-HER-2 biosimilars. The isotype antibodies did not have any impact. The MCF-7 cells and the patients’ breast epithelial cells did not show signs of responding to these immunotherapeutics in the conditions described. All the measurements were pursued in triplicates. All the data presented are representative for all studied. Treatment with the isotype antibodies did not have any statistically significant impact on the cancer cells’ death rates.

## Discussion

Herein, we have presented an entirely new paradigm for immunotherapy of cancer patients due to biomolecular engineering of synthetic molecules, which are capable for interfacing preventive and therapeutic immunities. It is a paradigm of redirecting, accelerating, amplifying vaccine-induced immunity therapy against cancer (RAAVIIT). It engages both arms of the patients’ immune system: innate and adaptive—acquired through vaccination against hepatitis B virus. HBsAg is the epitope uniquely different from all epitopes displayed on healthy human cells. Therefore, the risk of vaccination-induced immunity turning against the patients’ own cells, as autoimmune disease, is unlikely. Therefore, the approach presented herein offers a promise of being least iatrogenic.

This situation would be very different, if the vaccines would be ever developed against cancer cells. Even changes in the receptors’ coding sequences, e.g., epidermal growth factor 1 variant III (*EGFRvIII*) mutation deletion, which result in the mutated gene expression product as the truncated receptor, still retains large portions of the receptors’ wild type. In that scenario, the risk of an overlap with the non-mutated portion of the receptor and inclusion into the immune response to mount autoimmunity is higher. Therefore, linking the anti-body against a cancer unique molecule with the FDA already approved anti-viral vaccine seems to be a safer and faster approach.

Therefore, rather than developing vaccines imitating cancer molecules, so that the response would be directly targeting patients’ cells, the RAAVIIT approach may be more favorable. In RAAVIIT, the immunity is directed against a unique immunogen, which remains on the firm stand-by. The immune system is only activated either by HBsAg—vaccinating immunogen, or by the hot HBV, or by that immunogen linked with the anti-HER-2 antibody to redirect immune response.

Immunity developed in patients by vaccination relies upon generating polyclonal antibodies. Development of two immunotherapeutics: trastuzumab and pertuzumab, against the same target HER-2, is de facto an attempt of reconstructing the response of the natural immune system with polyclonal antibodies. Herein, we present a way to by-pass the need for developing multiple clones of antibodies against HER-2, but rather we use HBsAg, which serves as a lightning rod for attracting all the clones of antibodies generated by immunization. Therefore, it amplifies the therapeutic efficacy of the single clone of anti-HER-2 to the level equivalent to eliciting polyclonal antibodies. With this new strategy, we engage the entire immune system against cancer targets; thus greatly amplify the therapeutic efficacy.

Moreover, if ever developed, anti-cancer vaccines would have to be introduced against multiple cancers and assembled into a program, as now by the CDC for viruses and bacteria. Otherwise, development of the active, strong immune response by a patient suffering from a cancer, whose immune system is greatly compromised by the first line systemic chemotherapies, is hard. Furthermore, heterogeneity of cancer cells’ population might require multiple vaccines.

Furthermore, to develop an active immune response to the cancer vaccine may take a very long time. Passing time works in favor of cancer progression, but against patient’s chances of survival. The RAAVIIT secures the instantaneity of the immune response.

The FDA approved vaccines and tests determining the concentrations of the antibodies in blood of the patients are against *adw* type of hepatitis B virus. The interfacing molecules described herein are utilizing the patients’ immunity against those molecules. However, vaccinations against other types of the viruses may result in deviations of reading of the adw oriented tests from the real levels of immunity [29, 30]. Identifying, the specific virus type used for vaccination of the particular patient, administering the specific tests, and applying the specific HBsAg, should be the essential consideration, when administering the RAAVIIT to the patients, while assuring high therapeutic safety and efficacy.

The strategy of killing of the breast cancer cells designed in this work involves all three mechanisms of cell death: growth inhibition, apoptosis, and necrosis. This novel strategy also engages both arms of the immune system: innate and adaptive. The RAAVIIT’s biomolecules designed in this project have two major triggers of these mechanisms. First, the antibody portion of the RAAVIIT biomolecule, through the specific domains of the Fc, stimulate FcγR on immune cells. Moreover, the specific domains bind the C1q elements of the complement system, what leads to activation of the complement and initiating C3 action. Second, the HBsAg portions of the RAAVIIT biomolecules are triggering massive response of both the innate system and the active, vaccination induced adaptive immunity. This greatly accelerated and amplified response, over the passively administered monoclonal antibodies alone, explains great efficacy of RAAVIIT.

## Abbreviations

HER-2: human epidermal growth factor receptor 2
HBsAg: hepatitis B virus surface antigen
HBV: hepatitis B virus
VLP: virus like particle
CTL: cytotoxic lymphocyte
NKC: natural killer cell
NMR: nuclear magnetic resonance
XRFS: X-ray reflection fluorescence spectroscopy
FCM: fluorescent flow cytometry
FACS: fluorescent activated cell sorting
MFM: magnetic flow cytometry
MACS: magnetic activated cell sorting
RAAVIIT: redirected, accelerated, amplified, vaccination-induced, immunotherapy
AVEC: antibody vaccine engineered construct

## References

[1] Torre LA, Siegel RL, Ward EM, Jemal A (2016). Global cancer incidence and mortality rates and trends—an update. Cancer Epidemiol Biomarkers Prev..

[2] Slamon DJ, Clark GM, Wong SG, Levin WJ, Ulrich A (1987). Human breast cancer: correlation of relapse and survival with amplification of the HER-2/neu oncogene. Science.

[3] Pietras RJ, Pegram MD, Finn RS, Slamon DJ (1998). Remission of human breast cancer xenografts of therapy with humanized monoclonal antibody to HER-2 receptor and DNA-reactive drugs. Oncogene.

[4] Park JW, Stagg R, Lewis GD, Carter P, Maneval D, Slamon DJ, Jaffe H, Shepard HM (1992). Anti-p185HER2 monoclonal antibodies: biological properties and potential for immunotherapy. Cancer Treat Res.

[5] Figueroa-Magalhães MC, Jelovac D, Connolly RM, Wolff AC (2014). Treatment of HER2-positive breast cancer. Breast.

[6] http://www.fda.gov/drugs/developmentapprovalprocess/howdrugsaredevelopedandapproved/approvalapplications/therapeuticbiologicapplications/ucm080591.htm

[7] http://www.fda.gov/safety/medwatch/safetyinformation/safetyalertsforhumanmedicalproducts/ucm350817.htm; http://www.fda.gov/newsevents/newsroom/pressannouncements/ucm370393.htm

[8] Mendes D, Alves C, Afonso N, Cardoso F, Passos-Coelho JL (2015). The benefit of HER2-targeted therapies on overall survival of patients with metastatic HER2-positive breast cancer. Breast Cancer Res.

[9] Sliwkowski MX, Lofgren JA, Lewis GD, Hotaling TE, Fendly BM (1999). Nonclinical studies addressing the mechanism of action of Herceptin (Trastuzumab). Semin Oncol.

[10] Clifford A, Hudis MD (2007). Herceptin — Mechanism of action and use in clinical practice. N Engl J Med.

[11] Baselga J, Albanell J (2001). Mechanism of action of anti-HER2 monoclonal antibodies. Ann Oncol.

[12] Ahmad S, Gupta S, Kumar R, Varshney GC, Raghava GP (2014). Herceptin resistance database for understanding mechanism of resistance in breast cancer patients. Sci Rep.

[13] Rosenberg SA, Yang JC, Restifo NP (2004). Cancer immunotherapy: moving beyond current vaccines. Nat Med.

[14] Rosenberg SA (2004). Shedding light on immunotherapy for cancer. N Engl J Med.

[15] Daniels D, Grytdal S, Wasley A (2009). Surveillance for Acute viral hepatitis—United States, 2007. Morbidity and mortality weekly report. Surveillance summaries. 58:1–9. http://www.cdc.gov/mmwr/PDF/ss/ss5803.pdf 19478727

[16] Wolters GL, Kuijpers L, Kacaki J, Schuurs A (1976). Solid-phase enzyme-immunoassay for detection of hepatitis B surface antigen. J Clin Pathol.

[17] http://www.fda.gov/biologicsbloodvaccines/vaccines/approvedproducts/ucm398332.htm

[18] http://www.fda.gov/biologicsbloodvaccines/vaccines/approvedproducts/ucm376931.htm

[19] SK-BR-3 http://www.atcc.org/Products/All/HTB-30.aspx

[20] MCF-7 http://www.atcc.org/Products/All/HTB-22.aspx

[21] Malecki M, Hsu A, Truong L, Sanchez S (2002). Molecular immune-labelling with recombinant single-chain variable fragment (scFv) antibodies designed with metal-binding domains. Proc Natl Acad Sci.

[22] Malecki M, Szybalski W (2012). Isolation of single, intact chromosomes from single, selected ovarian cancer cells for in situ hybridization and sequencing. Gene.

[23] http://www.imgt.org/3Dstructure-DB/cgi/details.cgi?pdbcode=7637&Part=Chain&Chain=7637L

[24] http://www.imgt.org/3Dstructure-DB/cgi/details.cgi?pdbcode=7637&Part=Chain&Chain=7637H

[25] Valenzuela P, Gray P, Quiroga M, Zaldivar J, Goodman HM (1979). Nucleotide sequence of the gene coding for the major protein of hepatitis B virus surface antigen. Nature.

[26] Valenzuela P, Medina A, Rutter WJ, Ammerer G, Hall BD (1982). Synthesis and assembly of hepatitis B virus surface antigen particles in yeast. Nature.

[27] Hazra PP, Suriapranata I, Snyder WB, Subramani S (2002). Peroxisome remnants in Pex3 D cells and the requirement of Pex3p for interactions between the peroxisomal docking and translocation subcomplexes. Traffic.

[28] Kitagawa T, Aikawa T (1976). Enzyme coupled immunoassay of insulin using a novel coupling reagent. J Biochem.

[29] Heijtink RA, Bergen P, Melber K, Janowicz ZA, Osterhaus AD (2002). Hepatitis B surface antigen (HBsAg) derived from yeast cells used to establish an influence of antigenic subtype (adw2, adr, ayw3) in measuring the immune response after vaccination. Vaccine..

[30] Scheiblauer H, El-Nageh M, Diaz S, Nick S, Zeichhardt H (2010). Performance evaluation of 70 hepatitis B virus surface antigen (HBsAg) assays from around the world by a geographically diverse panel with an array of *HBSAG* genotypes and HBsAg subtypes. Vox Sang.

